# The Gender Non-affirmation from Sexual Partners Scale: Psychometric Evaluation and Correlates of a Brief Stigma Scale in a Sample Transgender and Gender Diverse Community Health Center Patients

**DOI:** 10.1007/s10461-026-05043-3

**Published:** 2026-03-17

**Authors:** Mari Tarantino, Asa E. Radix, Alexander Harris, Blanca C. Esquivel, Amiyah Guerra, Kenneth H. Mayer, Sari L. Reisner

**Affiliations:** 1 Department of Human Development and Family Science, Virginia Tech, 248 Wallace Hall, 291 W. Campus Drive, Blacksburg, VA 24061 USA; 2 Department of Epidemiology, Columbia University, New York, NY USA; 3 Callen-Lorde Community Health Center, New York, NY USA; 4 The Fenway Institute, Fenway Health, Boston, MA USA; 5 Department of Medicine, Beth Israel Deaconess Medical Center, Harvard Medical School, Boston, MA USA; 6 Department of Epidemiology, University of Michigan School of Public Health, Ann Arbor, MI USA; 7 Center for Social Epidemiology and Population Health, University of Michigan School of Public Health, Ann Arbor, MI USA; 8 Department of Epidemiology, Harvard T.H. Chan School of Public Health, Boston, MA USA

**Keywords:** Transgender persons, Social stigma, Sexual health, Community health centers

## Abstract

This study evaluated the psychometric performance and correlates of a 4-item GNSP scale among transgender, nonbinary, and gender diverse (TGD) adults. Data were collected in 2019–2020 via an electronic, self-reported cross-sectional survey of 2192 TGD adults accessing two U.S. LGBTQ+ community health centers. Participants reporting sex within the past 6 months (60.7%, *n* = 1463) were asked the 4-item GNSP scale. Analyses were internal consistency reliability (α), confirmatory factor analysis, and a multivariable logistic regression model of GNSP scale correlates (adjusted odds ratios [AOR], 95% confidence intervals [95% CI]). Mean age was 27 years (range = 18–71 years), 18.7% were people of color, and 33.6% nonbinary. 42.7% reported past 6-month GNSP. The GNSP scale showed good reliability (α = 0.78) loaded onto a single factor (eigenvalue = 2.45; variance explained = 48.7%). Higher odds of reporting GNSP included nonbinary identity (AOR = 1.63; 95% CI 1.51, 1.70), more past 6-month sexual partners (AOR = 1.19; 95% CI 1.17–1.21), a cisgender woman sexual partner (AOR = 1.51; 95% CI 1.42–1.61), a high school degree or less (AOR = 1.43; 95% CI 1.28–1.59), elevated alcohol use (AOR = 1.55; 95% CI 1.46–1.65), severe psychological distress (AOR = 1.80; 95% CI 1.68–1.92), penile sex with a condom (AOR = 1.74; 95% CI 1.59–1.90) or without a condom (AOR = 1.38; 95% CI 1.28–1.49), and current PrEP use (AOR = 1.42; 95% CI 1.25–1.61). Lower odds of reporting GNSP included older age vs. ages 18–24 (AORs range 0.65–0.75), transfeminine identity (AOR = 0.87; 95% CI 0.80–0.94), uninsured (AOR = 0.56; 95% CI 0.48–0.60), and in committed relationships (AOR = 0.36; 95% CI 0.33–0.40) or ethically non-monogamous relationships (AOR = 0.38; 95% CI 0.35–0.43). Gender non-affirmation from sexual partners was highly prevalent and may represent important intervention targets for sexual and mental health. The GNSP scale demonstrated good psychometric properties and is a promising tool for future TGD research.

**Supplementary Information:**

The online version contains supplementary material available at 10.1007/s10461-026-05043-3.

## Introduction

 In the United States (U.S.) as well as globally, transgender, nonbinary, and other gender diverse (TGD) people are heavily burdened by the HIV epidemic [1, 2]. A U.S. meta-analysis found laboratory-confirmed HIV prevalence of 3.2% (95% CI 1.4%–7.1%; *n* = 8 studies) in transgender men and 14.1% (95% CI 8.7%−22.2%; *n* = 13 studies) in transgender women [3]. HIV data are lacking among nonbinary people. Further, studies have found low rates of HIV testing and pre-exposure prophylaxis (PrEP) uptake, and high rates of condomless sex with multiple sexual partners among TGD populations in the U.S [3, 4], which highlights the need to understand the unique experiences and nuanced social contexts surrounding HIV burden and vulnerability among TGD people. The HIV epidemic for TGD people is driven by multiple factors, including stigma and social marginalization [5], and other highly prevalent health conditions such as depression, alcohol use, and gender-based violence including intimate partner violence [6]. These HIV-related inequities highlight the urgent need for research to elucidate the experiences, desires, and contexts of sexual health among TGD populations to inform optimal public health interventions.

Given HIV sexual acquisition and transmission behaviors occur in sexual partnerships, the HIV epidemic is inherently relational. Stigma, specifically gender non-affirmation from sexual partners, can have detrimental effects on the sexual health of TGD people. Gender affirmation refers to being recognized or affirmed in one’s gender identity or expression and is an important health determinant for TGD people [7, 8]. Studies demonstrate that TGD people may experience stigma from sexual and romantic partners [9–13]. TGD research has suggests that stress associated with experiencing gender non-affirmation from sexual partners can increase stigma-related HIV risk [8, 14]. Yet, few validated measures exist to assess this construct quantitatively.

Reisner et al. [14] developed and validated a brief 4-item stigma scale of gender non-affirmation for HIV research in a U.S. nationwide sample of transgender men who have sex with men, capturing experiences such as being misgendered or disrespected in sexual encounters. Higher gender non-affirmation from sexual partners was associated with adverse HIV-related outcomes including not recently testing for HIV and condomless sex in the last 6 months [14]. While this initial work has revealed insights into the role of gender-non affirmation in sexual partnership dynamics among transgender men, more research is needed to expand upon this study to other TGD populations, including validating the scale for transgender women and nonbinary people. Further, exploring gender identity differences in experiences of gender non-affirmation may yield valuable insights for future HIV prevention research and programming.

In the current study, we sought to evaluate the psychometric performance of the Gender Non-affirmation from Sexual Partners (GNSP) scale in a sample of TGD adults reporting a sexual partner in the last 6 months and identify correlates of gender-non affirmation in this study population. Our hypotheses were two-fold. First, we hypothesized that the GNSP scale would have good internal consistency reliability and load onto a single factor, representing the construct of gender non-affirmation from sexual partners. Second, in alignment with prior research [14], we anticipated HIV-related vulnerabilities, mental health and substance use, and violence would be associated with a higher odds of reporting gender non-affirmation from sexual partners.

## Method

### Participants and Procedures

Data used in this study were from the LEGACY Project, a multisite cohort consisting of TGD adults across two U.S. federally qualified community health centers in Boston, Massachusetts and New York, New York, with expertise in LGBTQ+ health. Participants were eligible if they were ages 18 years and older, had a gender identity that differed from the sex they were assigned from birth, and received primary care at either community health center (defined as at least one past 12-month primary care visit). An electronic self-reported cross-sectional survey was conducted between 2019 and 2020 with *N* = 2192 TGD adults to assess health and health needs. The detailed cohort methodology has been published elsewhere [15]. All research procedures were approved by Fenway Health Institutional Review Board.

### Measures

Measures were selected from prior TGD research and psychometrically validated instruments were utilized wherever possible. All reference groups were chosen conceptually based on existing psychosocial determinants research related to sexual health and partnerships.

#### Gender Non-affirmation from Sexual Partners (GNSP) Scale

Gender non-affirmation was measured using a brief 4 item GNSP measure of gender non-affirmation experienced from sexual partners in the last 6 months. The scale was previously developed in collaboration with TGD community members and validated with transgender men [14]. Participants reported past experiences of being misgendered, disrespected, questioned, or invalidated during/after sex in the last 6 months with responses options ranging from 0 = never to 3 = many times (see Supplemental Material, Table 1). Items were summed with scores ranging from 0 to 12, with higher scores indicating more frequent GNSP. Since more than half of the analytic sample did not indicate any GNSP scoring 0), gender non-affirmation was coded as a binary variable (1 = GNSP score ≥ 1, 0 = no reported GNSP).

**Table 1:** Sample characteristics of transgender, nonbinary, and gender diverse adults with a sexual partner in the last 6 months (*N* = 1463)

Gender non-affirmation from sexual partners scale, last 6 months (score range 0–12)		
Mean (SD)	1.53 (2.45)	–
Missing	129	–

	N	%
Any gender non-affirmation from sexual partners, last 6 months		
Yes	625	42.7
No	838	57.3
Age group in years		
18–24	461	31.5
25–29	386	26.4
30–39	438	29.9
40 and older	176	12.0
Missing	2	0.1
Racial identity^+^		
Asian	81	5.5
Pacific Islander	5	0.3
Native Hawaiian	1	0.1
Black/African American	116	7.9
White	1174	80.2
Hispanic/Latino	162	11.1
American Indian/Alaskan Native	22	1.5
Biracial/multiracial	189	12.9
Another racial/ethnic identity	53	3.6
Choose not to answer	8	0.5
Missing	8	0.6
Gender identity		
Transmasculine, transgender man	609	41.6
Transfeminine, transgender woman	361	24.6
Nonbinary, AMAB	105	7.1
Nonbinary, AFAB	386	26.3
Missing	2	0.2
Sexual orientation		
Monosexual straight	181	12.4
Monosexual lesbian/gay	232	15.9
Plurisexual/bisexual	962	65.8
Other	75	5.1
Missing	13	0.8
Education		
High school or less	157	10.7
Some college/vocational school	437	29.9
Completed bachelor’s degree	487	33.3
Some graduate school or more	357	24.4
Missing	25	1.7
Health insurance^+^		
Public	373	25.5
Private	982	67.1
Both private and public	47	3.2
Uninsured	61	4.2
Gender-affirming hormone use		
Currently taking	1209	82.6
Not taking, but want	186	12.7
Not taking, do not want	62	4.2
Missing	6	0.4
Number of sexual partners, last 6 months (range 1–100)		
Mean (SD)	2.64 (5.79)	–
Missing	18	–
Gender of sexual partners^+^		
Cisgender man	542	37.0
Cisgender woman	689	47.1
Transgender man	189	12.9
Transgender woman	187	12.8
Nonbinary	406	27.8
Relationship type^+^		
Single	252	17.2
Casual relationship	172	11.8
Committed relationship	697	47.6
Ethically non-monogamous	330	22.6
Missing	12	0.80
Transgender congruence scale (score range 1− 5)		
Mean (SD)	3.93 (0.84)	–
Missing	3	–
Intimate partner violence, lifetime (score range 0–11)		
Mean (SD)	1.59 (2.05)	–
Missing	6	–
Hazardous alcohol use (AUDIT-C SCORE ≥ 4)		
Yes	414	28.3
No	1025	70.1
Missing	24	1.6
Severe psychological distress, last 30 days (KESSLER-6 score ≥ 13)		
Yes	369	25.2
No	1093	74.7
Missing	1	0.07
Gender dysphoria (score range 0− 60)		
Mean (SD)	47.28 (7.87)	–
Missing	2	–
Gender euphoria (score range 1−10)		
Mean (SD)	8.33 (1.74)	–
Missing	10	–
HIV testing history		
Tested during lifetime	538	36.8
Tested within past 6 months	614	42.0
No testing history or unsure	294	20.1
Missing	17	1.1
Condomless penile-sex, last 6 months		
Penile sex with a condom	192	13.1
Penile sex without a condom	526	36.0
No penile sex	695	47.5
Missing	50	3.4
PrEP uptake		
Taken PrEP in lifetime	71	4.9
Currently taking PrEP	98	6.7
Never taken PrEP	1105	75.5
Missing	189	12.9

^+^Variables were select all that apply

*SD* standard deviation, *AFAB* Assigned Female at Birth, *AMAB* Assigned Male at Birth, *PrEP* Pre-Exposure Prophylaxis for Prevention of HIV Acquisition

#### Sociodemographics and Medical Gender Affirmation

Age was measured in years. Racial and ethnic identity questions allowed participants to choose all that apply from 10 categories (Asian, Pacific Islander, Native Hawaiian, Black/African American, White, Hispanic or Latino, American Indian/Alaskan Native, Biracial or Multiracial, another identity not listed, or choose not to answer). Race was recoded as a dichotomous variable (White and BIPOC categories), to optimize statistical power in the analysis. Participant gender identity was indicated by Transgender man/transmasculine (reference group), Transgender woman/transfeminine, and Nonbinary (e.g., genderqueer, gender nonconforming, other diverse identities). Sexual orientation was condensed to maintain power in analyses with categories of plurisexual/bisexual (the reference group) and straight/heterosexual. Education categories included the highest level of formal education achieved: A high school diploma or less, some college or vocational school, having a Bachelor’s degree (the reference group), or a Graduate Degree. Health insurance was measured by indicating Private (reference), public, or no health insurance. Gender-affirming hormone use was measured as one dimension of medical gender affirmation. Participants indicated whether they were currently taking hormones (reference group), not taking hormones but wanting to, or not taking hormones and not having an interest in doing so.

#### Sexual Partnerships

Three aspects of sexual partnerships were evaluated in this study. Participants reported the number of sexual partners in the last 6 months with whom they had any sexual contact. Gender of their partners was asked in a check any that apply format as Cisgender Male/Man; Cisgender Female/Woman; Transgender Man/transmasculine; Transgender Woman/transfeminine; Assigned Female at Birth (AFAB) Gender nonbinary, Genderqueer, Gender nonconforming; Assigned Male at Birth (AMAB) Gender nonbinary, Genderqueer, Gender nonconforming; Agender; Bigender; Another gender with a fill in option; and choose not to answer. These were re-coded as cisgender male/man, cisgender female/woman, transgender man/trans masculine, transgender woman/trans feminine, and nonbinary (inclusive of nonbinary, genderqueer, gender nonconforming, agender, bigender, and other). Participants indicated their current relationship status, also recorded with a check all that apply format: single/not dating, married/civil partnership, serious relationship, casually dating, polyamorous/poly, open relationship/non-monogamous, sleeping with someone but not dating, another status with a fill in option, and choose not to answer. For analyses, partnership status was recoded and condensed into four groups: casual relationships (reference), single, committed relationship, and ethically non-monogamous.

#### Internalized Transphobia and Violence

Internalized transphobia was measured using the 4-item Transgender Congruence Scale (TCS) [16], a validated measure of transgender individuals’ perceived (dis)comfort within their gender identity and expression. Participants responded to statements indicating their experiences within the past two weeks (i.e., “I am not proud of my gender identity”, “I have accepted my gender identity”). Response options ranged from 1 to 5. Items 2 and 3 were reverse scored. Items were summed with higher scores indicating more internalized transphobia.

Intimate partner violence was measured using 11 questions adapted from the World Health Organization multi-country study on women’s health and domestic violence [17] and the Transgender-Specific Intimate Partner Violence Scale [18]. Participants indicated “yes”, “no”, or “choose not to answer” following the prompt, “In your lifetime, did any of your partners do any of the following things to you?”. Example questions included, “Did a partner slap you, push you, shove you, or throw something at you that could hurt you?” and “Did a partner hide or destroy your hormones, prosthetics, chest binders, clothing, etc. related to gender transition?”. “Choose not to answer” responses were coded as missing. Items were summed to create a score ranging from 0 to 11, higher scores indicating more reported interpersonal violence. A score of 0 indicated no reported IPV.

#### Mental Health and Substance Use

Hazardous alcohol use was measured using an adapted version of the Alcohol Use Disorders Identification Test-Clinical (AUDIT-C) [19, 20]. Participants answered three questions about the amount and frequency of alcohol use, ranging from 0/Never to 4/Daily or almost daily with scores ranging from 0 to 12. A score of ≥ 4 was considered a positive screen for alcohol use disorder to be consistent with recommendations for TGD alcohol research [21]. Severe psychological distress was measured using the validated 6 item Kessler-6 Psychological Distress Scale [22] assessing respondents’ feelings of hopelessness, restlessness, and depression across the past 30 days. Items were scored from 0/none of the time to 4/all of the time, with scores ranging from 0 to 24. A score of ≥ 13 indicated clinically elevated severe psychological distress (classification accuracy = 0.92 for a serious mental illness) [22].

Gender dysphoria was measured using 12 adapted questions from the Utrecht Gender Dysphoria Scale [23]. Participants indicated how much they related to statements over the past 6 months, ranging from Agree completely/5 to Disagree completely/1. Example items included, “I would be happier if I had the primary or secondary sex characteristics of another gender”, and “I feel unhappy every time someone treats me as my assigned sex at birth”. Gender euphoria was measured using a 2-item subscale of the Transgender Congruence Scale [16]. Participants responded to questions about their experiences with gender over the past two weeks responding to a 5-point Likert scale ranging from Strongly disagree to Strongly Agree. Example items included, “I have accepted my gender identity” and “I am happy that I have the gender identity that I do”.

#### Sexual Health

Respondents answered two items asking about past 6-month HIV testing (i.e., “Have you been tested for HIV in the past 6 months?”) and lifetime testing (“Have you ever been tested for HIV?”). These variables were combined into a single variable with the mutually exclusive categories of HIV test within the last 6 months, HIV test in lifetime, or those who responded either “no” or “unsure” regarding their HIV testing history. Participants were also asked whether they engaged in sexual activity with a penis (“Of your [number] of partners in the past 6 months, how many did you have genital or anal sex with that included contact with a penis?”) and to indicate the number of partners with whom they had condomless penile sex in the past 6 months (recoded as no condomless penile-sex, yes condomless penile-sex, no penile-sex). Two questions were asked about lifetime and current PrEP use; these were combined into a single variable with mutually exclusive groups: ever take PrEP, currently taking PrEP, and never taken PrEP.

### Data Analysis

#### Statistical Procedures

Descriptive statistics were performed (mean, standard deviation, frequency, percentage) for the total sample. To assess the psychometric properties of the GNSP scale, we calculated internal consistency reliability (Cronbach’s alpha α) where 0.70–0.80 indicates good and 0.80–0.90 very good scale reliability [24]. Next, we conducted a confirmatory factor analysis, examining factor loadings, correlations, and fit indices to assess GNSP scale validity; calculating the eigenvalue proportion of variance explained; and visually inspecting the plotted eigenvalues from the scree plot.

To assess the sociodemographic and medical gender affirmation, sexual partnerships, stigma and violence, mental health and substance use, and sexual health correlates of GNSP, the primary analysis fit bivariate and multivariable logistic regression models (binary outcome of any GNSP yes/no). Odds ratios (OR), Adjusted Odds Ratios (AOR), and 95% Confidence Intervals (95% CI) were estimated. A secondary analysis was also conducted with GNSP scale scores (continuous outcome variable), fitting bivariate and multivariable linear regression models (Supplemental Material, Table 2), and estimating betas (b), adjusted betas (b), and 95% Confidence Limits (95% CL). For the secondary analysis, GNSP scale scores were z-scored (mean 0, standard deviation 1) to be interpretable in standard deviation units. For modeling, racial identity (BIPOC vs. White) was collapsed into a binary variable for statistical power. Statistical significance was determined at the alpha 0.05-level. Analyses were implemented in Statistical Package for the Social Sciences (SPSS), version 28.01.1. Confirmatory factor analyses were conducted using RStudio (version 2025.09.0 + 387).

**Table 2:**
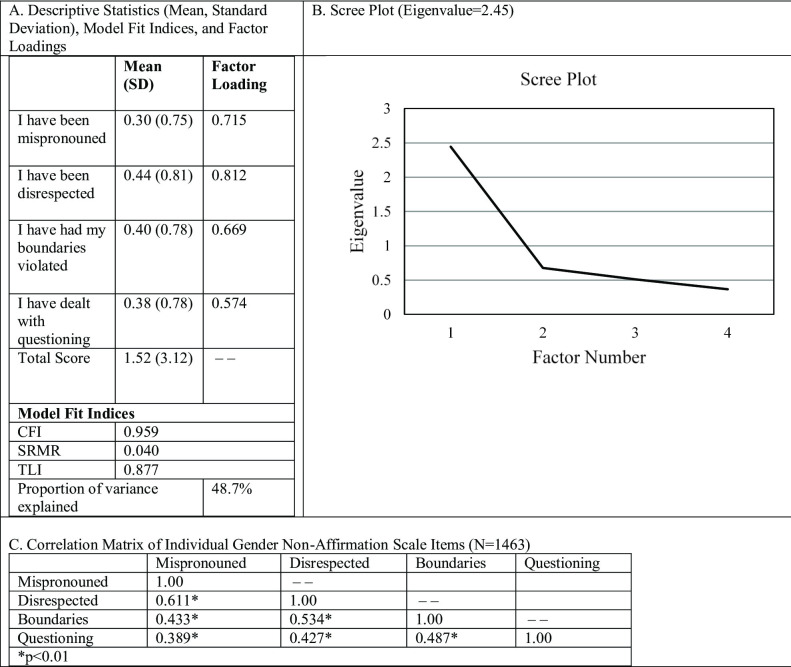
Psychometrics of gender Non-Affirmation sexual partners (GNSP) scale in the sample of transgender, nonbinary, and gender diverse adults with a sexual partner in the last 6 months

#### Missingness

The total sample consisted of *N* = 2912 participants. Cases were removed from further analysis if participants did not indicate any sexual partners in the last 6 months, as they were not asked the GNSP items due to skip logic (*N* = 729; 49.8% of the sample did not report a past 6-month sexual partnership and had structural missingness). The resulting data analytic sample was restricted to participants who reported one or more sexual partners within 6 months (*N* = 1463). A missing value analysis was then conducted on the GNSP scale and the independent variables. For GNSP, 91.2% of cases were complete with 8.8% missing values on GNSP. Little’s Missing Completely at Random (MCAR) test indicated data were not missing completely at random (*p* < 0.012). Multiple imputation using chained equations (MICE) was implemented with 25 imputations [25]. CFA and regression analyses were conducted pooling results according to Rubin’s rules [26]. Collinearity diagnostics were also assessed after fitting multivariable regression models to test for multicollinearity. All Variance Inflation Factor (VIF) values were within acceptable range (VIF < 4).

## Results

### Sample Demographics

The sample had a mean age of 29.6 years (SD = 8.7, range = 18–71 years), were predominantly White (80.2%), and bisexual/plurisexual (65.8%). Participants were transmasculine (41.6%), transfeminine (24.6%), and nonbinary (33.7%), and a majority had at least some college or vocational school experience (87.6%). Participants indicated being in a committed relationship, defined as having a boyfriend, girlfriend, or partner (47.6%), and were ethically non-monogamous (22.6%). Additional demographic information is presented in Table 1.

### Gender Non-affirmation from Sexual Partners Scale

#### GNSP Scale Score Distribution

The sample had an average GNSP score of 1.53 (SD=2.45, range = 0–12). Overall, 42.7% (*N* = 625) reported one or more experiences of gender non-affirmation from sexual partners in the last 6 months, indicated by at least one affirmative response to the four items: 13.5% reported a score of 1 (experiencing non-affirmation “once or twice”), 9.1% reported a score of 2, and 20.1% reported a score of 3 or higher.

#### Internal Consistency Reliability and Factor Analysis

Internal consistency reliability was good (α = 0.78) with item-total correlations (Pearson *r*) ranging from 0.39 to 0.61 (all *p* < 0.01; Table 2). In a confirmatory factor analysis, the 4-item scale loaded onto a single factor solution (eigenvalue = 2.45; variance explained = 48.7%; scree plot displayed in Table 2) representing the construct of gender non-affirmation. We also evaluated other CFA model fit indices including the Comparative Fit Index (CFI) ≥ 0.95 indicating a good fit (model CFI = 0.959), Tucker–Lewis Index (TLI) with values ≥ 0.90 indicating a good fit (model TLI was 0.877), and Standardized Root Mean Square Residual (SRMR) with the threshold of ≤ 0.08 for acceptable model fit (SRMR = 0.040). Taken together, the GNSP scale demonstrated acceptable model fit.

### Regression Models Estimating Correlates of Gender Non-affirmation

#### Primary Outcome Analysis

Table 3 presents results from the multivariable logistic regression model regressing any GNSP (score ≥ 1 vs. 0) on independent variables of interest, our primary analysis.

**Table 3:** Bivariate and multivariable logistic regression models of any reported gender non-affirmation from sexual partners in a sample of transgender, nonbinary, and gender diverse adults with a sexual partner in the last 6 months (*n* = 1463)

	Bivariate logistic regression models				Multivariable logistic regression model			
	OR	95% CI Lower	95% CI Upper	*p* value	AOR	95% CI Lower	95% CI Upper	*p* value
Constant	–	–	–	–	0.55	–	–	**< 0.001**
Age group in years (ref = 18–24)								
25–29	0.69	0.65	0.73	**< 0.001**	0.75	0.69	0.80	**< 0.001**
30–39	0.61	0.58	0.65	**< 0.001**	0.65	0.60	0.70	**< 0.001**
40 and older	0.63	0.59	0.68	**< 0.001**	0.69	0.62	0.76	**< 0.001**
Race (ref = BIPOC)								
White	0.87	0.83	0.91	**< 0.001**	1.06	0.99	1.13	0.115
Gender (ref = transmasculine)								
Transfeminine	0.98	0.93	1.03	0.404	0.87	0.80	0.94	**< 0.001**
Nonbinary	1.21	1.15	1.27	**< 0.001**	1.63	1.51	1.70	**< 0.001**
Sexuality (ref = plurisexual)								
Heterosexual	0.76	0.71	0.81	**< 0.001**	0.80	0.72	0.88	**< 0.001**
Lesbian/gay	0.68	0.64	0.72	**< 0.001**	0.84	0.78	0.90	**< 0.001**
Education (ref = bachelor’s degree)								
High school or less	1.47	1.36	1.59	**< 0.001**	1.43	1.28	1.59	**< 0.001**
Vocational school, some college	1.46	1.39	1.54	**< 0.001**	1.14	1.06	1.23	**< 0.001**
Graduate degree or higher	1.08	1.02	1.14	**0.008**	1.16	1.08	1.25	**< 0.001**
Insurance (ref = private)								
Public	1.18	1.13	1.24	**< 0.001**	1.05	0.98	1.12	0.201
Uninsured	1.00	0.90	1.11	0.963	0.56	0.48	0.60	**< 0.001**
Hormone use (ref = taking hormones)								
Not taking hormones, but want	1.35	1.27	1.44	**< 0.001**	1.27	1.17	1.39	**< 0.001**
Not taking hormones, do not want	0.81	0.72	0.90	**< 0.001**	0.81	0.702	0.90	**0.002**
Number of sexual partners, last 6 months (continuous)	1.27	1.26	1.29	**< 0.001**	1.19	1.17	1.21	**< 0.001**
Sexual partner gender (ref = cisgender man)								
Cisgender woman	1.08	1.04	1.13	**< 0.001**	1.51	1.42	1.61	**< 0.001**
Transgender man	0.92	0.86	0.98	**0.006**	0.71	0.65	0.78	**< 0.001**
Transgender woman	1.12	1.05	1.19	**< 0.001**	0.83	0.76	0.90	**< 0.001**
Nonbinary	1.20	1.14	1.26	**< 0.001**	1.07	1.00	1.15	0.070
Relationship status (ref = casual relationships)								
Single	0.74	0.68	0.80	**< 0.001**	1.00	0.90	1.11	0.972
Committed	0.24	0.22	0.26	**< 0.001**	0.36	0.33	0.40	**< 0.001**
Ethically non-monogamous	0.36	0.34	0.39	**< 0.001**	0.38	0.35	0.43	**< 0.001**
Transgender congruence (continuous)	0.77	0.75	0.79	**< 0.001**	0.98	0.92	1.04	0.436
Interpersonal violence (continuous)	1.18	1.17	1.19	**< 0.001**	1.12	1.10	1.14	**< 0.001**
Hazardous alcohol use	1.67	1.60	1.75	**< 0.001**	1.55	1.46	1.65	**< 0.001**
Severe psychological distress	2.03	1.93	2.13	**< 0.001**	1.80	1.68	1.92	**< 0.001**
Gender dysphoria (continuous)	1.021	1.018	1.023	**< 0.001**	1.023	1.018	1.027	**< 0.001**
Gender euphoria (continuous)	0.88	0.87	0.89	**< 0.001**	0.93	0.91	0.96	**< 0.001**
HIV testing history (ref = never tested)								
Tested in lifetime	0.84	0.79	0.89	**< 0.001**	0.91	0.83	0.99	**0.025**
Tested within past 6 months	1.73	1.63	1.83	**< 0.001**	1.30	1.20	1.42	**< 0.001**
Condomless penile sex, last 6 months (ref = no engagement in penile sex)								
Penile sex with a condom	1.95	1.83	2.09	**< 0.001**	1.74	1.59	1.90	**< 0.001**
Penile sex without a condom	2.00	1.91	2.09	**< 0.001**	1.38	1.28	1.49	**< 0.001**
PrEP use history (ref = never taken PrEP)								
Taken in lifetime	1.43	1.29	1.59	**< 0.001**	0.84	0.73	0.96	**0.009**
Currently taking PrEP	3.38	3.07	3.71	**< 0.001**	1.42	1.25	1.61	**< 0.001**

Bolded text indicates statistical significance *p* < .05

Outcome variable was any gender non-affirmation from sexual partners in the last 6 months using the GNSP scale: yes (score ≥ 1) vs. no (score 0). Data were multiply imputed (25 imputations) and results were pooled according to Rubin’s rules

*OR* odds ratio, *AOR* adjusted odds ratio, *95% CI* 95% confidence interval, *PrEP* Pre-Exposure Prophylaxis for Prevention of HIV Acquisition, *BIPOC* Black, Indigenous, and Other People of Color

^+^Fit statistics for the multivariable model: Standard error = 0.87, F-change = 247.97 (35, 27186), p value < 0.001

Hosmer and Lemeshow test: Chi square = 36.15, df = 8, p value < 0.001; Cox & Snell = 0.21

**Decreased Odds of Reporting GNSP**. In the multivariable model, sociodemogaraphic characteristics associated with lower odds of reporting any GNSP included older age groups vs. 18–24 year group (ages 25–29 years: AOR = 0.75, 95% CI 0.69, 0.80; ages 30–39 years: AOR = 0.65, 95% CI 0.60, 0.70; 40+ years: AOR = 0.69, 95% CI 0.62, 0.76); transgender woman/transfeminine vs. transgender man identity (AOR = 0.87, 95% CI 0.80, 0.94); lesbian/gay (AOR = 0.84, 95% CI 0.78, 0.90) and heterosexual (AOR = 0.80, 95% CI 0.72, 0.88) vs. plurisexual identity; no health insurance compared to private insurance (AOR = 0.56, 95% CI 0.48, 0.60). Also associated with lower odds of any GNSP were not taking or wanting to take gender-affirming hormones vs. taking hormones (AOR = 0.81, 95% CI 0.70, 0.90). Within sexual partnership correlates, compared to reporting a cisgender man sexual partner, participants with transgender man (AOR = 0.71, 95% CI 0.65, 0.78) or transgender woman (AOR = 0.83, 95% CI 0.76, 0.90) as sexual partners had a lower odds of reporting any GNSP, and those in either a committed relationship (AOR = 0.36, 95% CI 0.33, 0.40) or ethically non-monogamous (AOR = 0.38, 95% CI 0.35, 0.43) had a lower odds of reporting any GNSP. Greater gender euphoria scores were associated with a decreased odds of reporting any GNSP (AOR = 0.93, 95% CI 0.91, 0.96).

**Increased Odds of Reporting GNSP**. Sociodemographic variables associated with increased odds of any GNSP were nonbinary vs. transmasculine identity (AOR = 1.63, 95% CI 1.51, 1.70) and each educational attainment level vs. Bachelor’s degree (high school or less: AOR = 1.43, 95% CI 1.28, 1.59; vocational/some college: AOR = 1.14, 95% CI 1.06, 1.23; graduate degree or higher: AOR = 1.16, 95% CI 1.08, 1.25). Participants not taking hormones but wanting to had higher odds of reporting any GNSP (AOR = 1.27, 95% CI 1.17, 1.39). Sexual relationship and behavior factors associated with elevated odds of any GNSP were having a greater number of past 6-month sexual partners (AOR = 1.19, 95% CI 1.17, 1.21), a cisgender woman vs. cisgender man sexual partnership (AOR = 1.51, 95% CI 1.42, 1.61), and reporting both penile sex with condoms (AOR = 1.74, 95% CI 1.59, 1.90) and without condoms (AOR = 1.38, 95% CI 1.28, 1.49) vs. no penile sex. Psychosocial correlates associated with increased odds of GNSP were interpersonal violence (AOR = 1.12, 95% CI 1.10, 1.14), hazardous alcohol use (AOR = 1.55, 95% CI 1.46, 1.65), severe psychological distress (AOR = 1.80, 95% CI 1.68, 1.92), and higher gender dysphoria scores (AOR = 1.023, 95% CI 1.018, 1.027).

**Mixed Effects**. PrEP use and HIV testing both showed mixed patterns related to reporting GNSP. Compared to the reference group (never taken PrEP), lifetime PrEP use was associated with lower odds of experiencing GNSP (AOR = 0.84, 95% CI 0.73, 0.96), whereas current PrEP use was associated with increased odds (AOR = 1.42, 95% CI 1.25, 1.61). Similarly, lifetime HIV testing was associated with lower odds of any GNSP (AOR = 0.91, 95% CI 0.83, 0.99), while past 6-month HIV testing was associated with an increased odds (AOR = 1.30, 95% CI 1.20, 1.42), both relative to no history of HIV testing.

#### Secondary Outcome Analysis

Our secondary analysis modeled the frequency of GNSP using linear regression models with GNSP scale scores (z-scored) as the outcome variable (Supplemental Material, Table 2). Findings on the frequency of GNSP experienced were comparable to the primary analysis.

## Discussion

This study of TGD adults reporting a sexual partner in the last 6 months found that the GNSP Scale demonstrated sound psychometric properties. As hypothesized, the scale had good reliability and validity, loading onto a single-factor solution representing the construct of sexual partner gender non-affirmation. Extending prior research validating the GNSP Scale with transmasculine individuals, the current study of TGD people with diverse gender identities, including transgender women, transgender men, and nonbinary people, offers a promising tool for future TGD research endeavors. Stigma, specifically gender non-affirmation from sexual partners, was ubiquitous in the sample with 42.7% reporting one or more experiences of gender non-affirmation from a sexual partner in the last 6 months. Consistent with our hypotheses, and corroborating prior research [14], several self-reported HIV-related and mental health indicators, including engaging in penile sex within the past 6 months, having more sexual partners, currently taking PrEP, reporting hazardous alcohol use, and reporting past 30-day severe psychological distress were associated with higher odds of reporting any sexual partner gender non-affirmation. Findings highlight an under researched dimension of some TGD individuals’ lived experience in sexual relationships and the health-related sequelae of gender non-affirmation, a potential contributor to trans-specific minority stress [27].

Several sexual health-related variables were associated with reported experiences of GNSP. First, having a greater number of past 6-month sexual partners was associated with higher odds of gender-non affirmation from partners. It is possible that having more partners increases the likelihood that a TGD person is exposed to a sexual partner who is not gender-affirming. Notably, ethical non-monogamy was associated with lower odds of GNSP. This finding is important in dispelling inaccurate, stigmatizing stereotypes that conflate ethical non-monogamy with increased sexual risk [28], and underscoring that relationship structure and philosophy (e.g., ethical non-monogamy) is not synonymous with having more sexual partners [28]. Additional research is recommended to explore gender non-affirmation across different types of sexual partnerships, including mixed-methods research to elucidate gender non-affirmation in the context of casual, committed, and other sexual partnerships.

Other HIV-specific correlates associated with an increased probability of gender non-affirmation included engaging in penile sex, which was reported by almost half (49.1%) of the sample. Specifically, compared to participants reporting no penile sex, those reporting penile sex without a condom and penile sex with a condom had higher odds of experiencing gender non-affirmation from partners. It is possible that engagement in penile sex increases the potential for experiencing gender non-affirmation in the context of heteronormative sexual scripts shaping sexual behaviors surrounding penile/vaginal or penile/anal penetrative intercourse [29]. Interpretation of our findings is complicated by the heterogeneous reference group of no penile sex, which includes participants who are partnered to someone with a flesh penis not engaging in penile sex and those whose partnerships do not include someone with a flesh penis at all. However, this association has not, to our knowledge, yet been studied empirically and provides a unique direction for future exploratory research.

With regard to HIV prevention behaviors, 42% of our sample was tested for HIV in the last 6 months, 36% engaged in condomless penile sex in that timeframe, and 6.7% reported current PrEP use. Engagement in past 6-month HIV testing and current PrEP use were both associated with increased odds of gender non-affirmation from sexual partners. One possible reason for this association could derive from relationship dynamics at play during sexual encounters where gender non-affirmation occurs, and which may also be associated with HIV testing and PrEP use, therefore confounding the observed association in this study. For example, if someone perceives their sexual relationship as coercive, disrespectful, or unsafe—consistent with our finding of a significant association of IPV with GNSP—they may also seek out additional health promotion strategies like being tested more frequently for HIV or initiating PrEP, particularly if negotiating HIV prevention behaviors (e.g., condoms) is impacted by IPV. Recent literature identified several correlates of increased HIV testing for transgender women, including engagement in casual sex and experiencing harassment [30] supporting this potential explanation. TGD individuals may also use PrEP to mitigate the potential risk of violence, particularly in situations where they are unable to negotiate condom use or make the decision to engage in condomless sex due to external circumstances (e.g., IPV, transactional sex encounters for more money [31]). Given low overall PrEP uptake (75.5% had never used PrEP) and suboptimal recent HIV testing rates, future research is necessary to examine sexual partner gender non-affirmation and HIV prevention behaviors, including barriers to PrEP access.

We found that psychological distress and gender dysphoria, interconnected psychosocial constructs (Pease et al., 2023), were associated with a higher odds of reporting any non-affirmation. Recent work identified trans-specific microaggressions, like having to negotiate one’s gender identity and partners minimizing one’s gender identity, as common within the romantic relationships of TGD individuals [32]. Related research has found similar LGBTQ+ microaggressions were associated with negative mental health outcomes [33]. Our findings highlight gender non-affirmation as a trans-specific stressor likely contributing to minority stress-related pathways among TGD individuals.

Several sociodemographic characteristics were associated with reporting GNSP. Compared to ages 18–24, all older age groups had lower odds of experiencing gender non-affirmation from sexual partners. A wealth of prior literature has acknowledged that during the developmental period of emerging adulthood in ages 18–24 years [34], some individuals engage in heightened sexual risk behaviors [35]. This finding could also indicate that the sexual relationships of TGD people after emerging adulthood may be more affirming than sexual relationships earlier in adulthood. This interpretation is consistent with prior scholarship that for some marginalized individuals, particularly those who are sexually minoritized, earlier queer relationships compared to those later in life, were more *homonormative* such that partners embraced relationship roles and practices aligned with what was socially expected rather than what was authentic [36]. Additional research is recommended to understand the developmental context of gender non-affirmation for TGD emerging and young adults, including developmental determinants of more affirming partnerships for interventions to enhance gender affirmation.

Additionally, identifying as a transgender woman vs. a transgender man was associated with lower odds of reporting any GNSP. This finding was surprising given the increased risk that transfeminine individuals face for encountering IPV [18] and stigmatization [37]. However, the current study found that experiencing lifetime IPV was also independently associated with higher odds of experiencing gender non-affirmation, even with adjustment for gender identity. Lifetime IPV increases the likelihood of subsequent violence and re-victimization [38], thus it may be that TGD people with a history of IPV are more likely to be in sexual partnerships where gender non-affirmation occurs. Further, gender affirmation may be a unique dimension or form of trans-specific IPV [39]. Additional research is warranted to explore relationship violence and tactics of control in TGD people, including the role of gender non-affirmation by perpetrators. Further, the GNSP scale was initially created using qualitative survey data from transgender men who have sex with men (trans MSM) [14]. Thus, there could be specific characteristics of experiencing gender non-affirmation as a transfeminine person that are not captured in the current GNSP scale (i.e., transactional sex work) which may account for the association of GNSP with transfeminine identity. Refinement of the scale for transfeminine contexts may be warranted.

### Limitations and Strengths

We encourage interpretation of findings alongside the study limitations. First, data were drawn from a cohort of adult TGD primary care patients from LGBTQ+ community health centers in the New York and Massachusetts, United States. Findings may not generalize to other TGD populations or geographic settings and require replication in other contexts, such as TGD people not engaged in primary care and with greater geographic diversity. To maintain statistical power we collapsed or recoded some categorical variables (e.g., racial identity). We acknowledge that in doing so, there could be important subgroup differences that are not captured in our study. Future research with larger samples is recommended to maintain more complexity within social context and experiences of TGD adults in underrepresented groups. Relatedly, our data analytic sample was restricted to the subsample of participants who reported at least one sexual partner within the past 6 months which may have introduced selection bias. Second, the cross-sectional study design precludes causal inference, and we were not able to evaluate other psychometric properties of the scale such as test re-test reliability. Future longitudinal research is needed to make more causal claims about the determinants of gender non-affirmation and associations with health outcomes. Further research might also explore gender non-affirmation with other gender-affirming pathways, such as uptake of affirming surgical procedures.

Third, there are limitations relating to measurement of sexual and romantic partnerships that future research can address. For example, our measure for IPV did not specify whether behaviors included in the measure (i.e., slapping, name-calling partners) were non-consensual or consensual behavior as in the case of consensual BDSM (Bondage & Discipline, Dominants & Submission, and Sadism & Masochism) relationships. Future work could revise this language to avoid conflation of wanted consensual partnered activities and non-consensual partner violence. Additionally, variables related to sexual partnerships did not distinguish whether those relationships were concurrent or sequential. More detailed inquiry into the timelines, nature, and overlap of intimate partnerships could provide more insight into the associations between gender non-affirmation from partners and the relational contexts in which they are situated.

Regarding sexual health variables, while we intentionally examined penile sex and condom use in this analysis due to empirical support associating condom use differences with HIV risk [40], future work should consider a broader range of sexual behaviors and sexual health promotion strategies to be inclusive of diversity within TGD intimate partnerships. Some measures (e.g., lifetime HIV testing, PrEP use), relied on self-report and may be subject to recall bias. Finally, we were unable to consider HIV serostatus in this analysis, which may affect associations with HIV testing and PrEP. This study also had several strengths including the first validation of the GNSP scale for TGD populations beyond transmasculine individuals. We recognize that some effect sizes are small; however, the findings still offer valuable insights into the practical implications from this research. This work paves the way for subsequent research to examine the mechanisms underlying gender non-affirmation within the sexual partnerships of TGD people, and provides a strong foundation from which to use this measure in future research on HIV acquisition risk, mental health, and sexual partnerships.

### Future Directions and Conclusion

This study provides a novel psychometrically sound measure for researchers to continue exploring how relational contexts influence TGD sexual and mental health. Developing a more holistic, relational understanding of TGD intimate partnerships [41, 42] may inform future HIV prevention and sexual health intervention efforts. While gender non-affirmation from sexual partners was highly prevalent, the absence of GNSP (53.3% of the sample did not report these experiences) is also important to note. This counters deficit-based narratives that assume sexual relationships for TGD people are uniformly stigmatizing and provides evidence of relational resilience, supportive partners, and affirming sexual contexts. Future research could explore the role of intimate relationship dynamics, past relational histories, and relationship power as these relate to gender non-affirmation within TGD sexual partnerships. Future work could also consider dyadic perspectives of both or all partners to understand the mechanisms underlying both experiencing and generating gender non-affirmation within sexual partnerships. Finally, we encourage these findings being applied to future HIV prevention and intervention research among gender minoritized populations, particularly efforts which center on the intimate relationship experiences and their role in HIV acquisition risk for TGD people.

## Supplementary Information

Below is the link to the electronic supplementary material.

Supplementary Material 1

## Data Availability

Data from this study are not available publicly. Data usage interest can be directed to PI Dr. Sari Reisner at sreisner@umich.edu.
