# Supplementary material for: The Gender Non-affirmation from Sexual Partners Scale: Psychometric Evaluation and Correlates of a Brief Stigma Scale in a Sample Transgender and Gender Diverse Community Health Center Patients
Source: AIDS Behav. 2026 Mar 17;30(7):2174–87. doi: 10.1007/s10461-026-05043-3 (PMC13400690; doi:10.1007/s10461-026-05043-3)
Supplement: Supplementary file 1 — Supplementary Material 1 [file 10461_2026_5043_MOESM1_ESM.docx]

**SUPPLEMENTAL MATERIAL**

**Supplemental Table 1.** Gender Non-Affirmation from Sexual Partners (GNSP) Scale.

| In the past 6 months, please indicate how often the following has happened to you in the context of a sexual encounter. | | | | | |
| --- | --- | --- | --- | --- | --- |
|  | Never (0) | Once or twice (1) | A few  times (2) | Many times (3) | Choose not to answer (99) |
| I have been mis-pronouned/misgendered during or after sex. |  |  |  |  |  |
| I have been disrespected by having my body referred to by words/terms that I felt uncomfortable with. |  |  |  |  |  |
| I have crossed boundaries sexually that I later felt uncomfortable with or ashamed about in order to validate my gender identity or expression in the sexual encounter. |  |  |  |  |  |
| I have dealt with a sexual partner questioning their sexual orientation after having sex with me (e.g., a gay guy questioning his identity, or a heterosexual woman questioning her identity). |  |  |  |  |  |

Responses to the four GNSP scale items are summed, with scores ranging from 0-12, and higher scores indicate more frequent GNSP. In the current study, 8.8% of participants responded, “Choose not to answer.” These responses were coded as missing and were multiply imputed for analyses. The GNSP measure was previously developed in collaboration with transgender, nonbinary, and gender diverse (TGD) community members and validated with transgender men. Original source citation: Reisner SL, Moore CS, Asquith A, Pardee DJ, Mayer KH. Gender non-affirmation from cisgender male partners; Development and validation of a brief stigma scale for HIV research with transgender men who have sex with men. AIDS & Behavior 2020; 24(1): 331-343. doi: 10.1007/s10461-019-02749-5. The current study expands psychometric validation of the GNSP scale to a TGD sample with diverse gender identities and sexual partner genders.

**Supplemental Table** **2.** Bivariate and Multivariable Linear Regression Models of Gender Non-Affirmation from Sexual Partners in a Sample of Transgender, Nonbinary, and Gender Diverse Adults with a Sexual Partner in the Last 6 Months (n=1463).

|  | Bivariate  Linear Regression Models | | | | Multivariable  Linear Regression Model | | | |
| --- | --- | --- | --- | --- | --- | --- | --- | --- |
|  | Crude Beta | 95% CL Lower | 95% CL Upper | p-value | 95% CL Lower | 95% CL Upper | Adjusted Beta | p-value |
| (Constant) | -- | -- | -- | -- | –0.09 | 0.025 | –0.32 | 0.272 |
| **Age Group in Years** (ref=18–24) | | | | | | | | |
| 25–29 | –0.232 | –0.259 | –0.204 | **<0.001** | –0.277 | –0.218 | –0.111 | **<0.001** |
| 30–39 | –0.198 | –0.225 | –0.171 | **<0.001** | –0.305 | –0.243 | –0.126 | **<0.001** |
| 40+ | –0.218 | –0.253 | –0.182 | **<0.001** | –0.316 | –0.235 | –0.091 | **<0.001** |
| **Race** (ref=BIPOC) | | | | | | | | |
| White | –0.152 | –0.175 | –0.128 | **<0.001** | –0.008 | 0.044 | 0.008 | 0.168 |
| **Gender** (ref=Transmasculine) | | | | | | | | |
| Transfeminine | –0.066 | –0.093 | –0.039 | **<0.001** | –0.144 | –0.085 | –0.048 | **<0.001** |
| Gender Nonbinary | 0.069 | 0.045 | 0.093 | **<0.001** | 0.141 | 0.198 | 0.081 | **<0.001** |
| **Sexuality** (ref=Plurisexual) | | | | | | | | |
| Heterosexual | –0.114 | –0.147 | –0.081 | **<0.001** | –0.120 | –0.045 | –0.026 | **<0.001** |
| Lesbian/Gay | –0.191 | –0.221 | –0.162 | **<0.001** | –0.126 | –0.067 | –0.036 | **<0.001** |
| **Education** (ref=Bachelor’s Degree) | | | | | | | | |
| High School or Less | 0.238 | 0.200 | 0.276 | **<0.001** | 0.054 | 0.137 | 0.028 | **<0.001** |
| Vocational School, Some College | 0.226 | 0.200 | 0.252 | **<0.001** | –0.006 | 0.050 | 0.010 | 0.124 |
| Graduate Degree or Higher | 0.120 | 0.093 | 0.148 | **<0.001** | 0.094 | 0.150 | 0.054 | **<0.001** |
| **Insurance** (ref=Private) | | | | | | | | |
| Public | 0.123 | 0.100 | 0.147 | **<0.001** | 0.020 | 0.073 | 0.021 | **<0.001** |
| Uninsured | 0.137 | 0.085 | 0.189 | **<0.001** | –0.129 | –0.018 | –0.015 | **0.009** |
| **Hormone Use** (ref=Taking Hormones) | | | | | | | | |
| Not Taking Hormones, But Want | 0.145 | 0.113 | 0.176 | **<0.001** | 0.039 | 0.108 | 0.024 | **<0.001** |
| Not Taking Hormones, Do Not Want | –0.230 | –0.283 | –0.177 | **<0.001** | –0.231 | –0.124 | –0.038 | **<0.001** |
| **Number of Partners, Last 6 Months** (continuous) | 0.037 | 0.036 | 0.039 | **<0.001** | 0.019 | 0.023 | 0.141 | **<0.001** |
| **Partner Gender** (ref=Cisgender man) | | | | | | | | |
| Cisgender Woman | 0.041 | 0.020 | 0.063 | **<0.001** | 0.165 | 0.213 | 0.094 | **<0.001** |
| Transgender Man | 0.013 | –0.019 | 0.045 | 0.423 | –0.15 | –0.086 | –0.041 | **<0.001** |
| Transgender Woman | 0.039 | 0.008 | 0.070 | **0.015** | –0.174 | –0.107 | –0.049 | **<0.001** |
| Gender Nonbinary | 0.005 | –0.020 | 0.029 | 0.700 | –0.062 | –0.008 | –0.016 | **0.010** |
| **Relationship Status** (ref=Casual relationships) | | | | | | | | |
| Single | –0.067 | –0.106 | –0.029 | **<0.001** | 0.021 | 0.100 | 0.022 | **0.003** |
| Committed | –0.673 | –0.706 | –0.640 | **<0.001** | –0.496 | –0.426 | –0.230 | **<0.001** |
| Ethically Mon–monogamous | –0.478 | –0.514 | –0.442 | **<0.001** | –0.39 | –0.313 | –0.153 | **<0.001** |
| **Transgender Congruence** (continuous) | –0.140 | –0.153 | –0.128 | **<0.001** | –0.056 | –0.008 | –0.026 | **0.009** |
| **Interpersonal Violence** (continuous) | 0.120 | 0.115 | 0.125 | **<0.001** | 0.072 | 0.083 | 0.157 | **<0.001** |
| **Hazardous Alcohol Use** | 0.146 | 0.123 | 0.169 | **<0.001** | 0.001 | 0.048 | 0.011 | **0.043** |
| **Severe Psychological Distress** | 0.404 | 0.380 | 0.428 | **<0.001** | 0.229 | 0.281 | 0.110 | **<0.001** |
| **Gender Dysphoria**  (continuous) | 0.014 | 0.012 | 0.015 | **<0.001** | 0.011 | 0.014 | 0.096 | **<0.001** |
| **Gender Euphoria** (continuous) | –0.065 | –0.071 | –0.059 | **<0.001** | –0.026 | –0.003 | –0.025 | **0.011** |
| **HIV Testing History** (ref=Never Tested) | | | | | | | | |
| Tested in Lifetime | –0.034 | –0.062 | –0.005 | **0.021** | 0.026 | 0.091 | 0.028 | **<0.001** |
| Tested Within Past 6 Months | 0.365 | 0.337 | 0.393 | **<0.001** | 0.221 | 0.288 | 0.127 | **<0.001** |
| **Condomless Penile Sex** (ref=No Engagement in Penile Sex) | | | | | | | | |
| Penile Sex With a Condom | 0.296 | 0.264 | 0.328 | **<0.001** | 0.193 | 0.262 | 0.079 | **<0.001** |
| Penile Sex Without a Condom | 0.338 | 0.315 | 0.360 | **<0.001** | 0.164 | 0.221 | 0.094 | **<0.001** |
| **PrEP Use History** (ref=Never Taken PrEP) | | | | | | | | |
| Taken in Lifetime | 0.245 | 0.194 | 0.296 | **<0.001** | –0.121 | –0.024 | –0.017 | **0.004** |
| Currently Taking PrEP | 0.524 | 0.481 | 0.566 | **<0.001** | 0.096 | 0.186 | 0.037 | **<0.001** |

Outcome variable: Continuous GNSP scale scores; higher scores indicate greater frequency of gender non-affirmation from sexual partners in the last 6 months.

Model Fit Statistics for Multivariable Linear Regression: R=0.492, R^2^=0.242, Std. Error=0.871, R-Square Change=0.242, p-value F-Change=<0.001.

BIPOC=Black, Indigenous, and Other People of Color. PrEP=Pre-Exposure Prophylaxis
